# A simplified guide ruler from numeric table method in doing rotational osteotomy

**DOI:** 10.1186/1471-2474-9-87

**Published:** 2008-06-16

**Authors:** Chen-Kun Liaw, Rong-Sen Yang, Sheng-Mou Hou, Tai-Yin Wu, Chiou-Shann Fuh

**Affiliations:** 1 Department of Orthopaedics, Tao-Yuan General Hospital, Tao-Yuan city, Taiwan; 2 Department of Orthopaedics, College of Medicine, National Taiwan University & Hospital, Taipei city, Taiwan; 3 Taipei City Hospital, Renai Branch, Taipei city, Taiwan; 4 Institute of Computer Science and Information Engineering, National Taiwan University, Taipei city, Taiwan

## Abstract

**Background:**

Čobeljić et al. recently reported a numeric table method to provide precise rotational osteotomy which is a well established orthopaedic procedure. The numeric table requires four pages in length that is rather inconvenient during performing an osteotomy operation.

**Methods:**

We thus develop our own method by summarizing the data of the four-page table into a small ruler, which is easy to carry and use in operation room. An electrical version of this ruler is also available. We also build a computer model to verify Čobeljić et al. method.

**Results:**

The error of Čobeljić et al. is between -37% to 16% (mean ± SD = -6% ± 9%). We verify our ruler by calculating the absolute difference between our method and that of Čobeljić et al. The difference is less than 0.1 mm.

**Conclusion:**

Our ruler is convenient for practical use for the rotational osteotomy procedure with equal precision. Further clinical verification is needed to justify its real significance.

## Background

Rotational osteotomy is commonly used for correction of the deformity in the orthopaedic practice, e.g. malunited fractures, congenital or developmental deformities. Many methods have been reported to determine the proposed correcting rotation angle. Among them a method proposed by Hoffer et al. requires two Kirschner wires or two Steinmann pins as guide of correction during operation [1]. However, surgeons usually find difficulty in correctly positioning these pins, especially when the planned correction angles are not round values (10°, 20°, 30°...). Another method by Lasserre et al. needs calculations and is inconvenient for use [2]. Fait et al. proposed a similar method that allows correction angle in steps (10°, 20°, 30°...), whereas it is unacceptable in an operation needing precise correction [3].

Čobeljić et al. recently published a method using a huge table of four printed pages [4]. Therefore surgeons can find the assumed correction chord distance in that table by attempted correction angle and bone diameter, and then perform the osteotomy. Such a method had a satisfactory precision and no sterilization is needed, except inconvenient to use a huge four-page table during the real practice.

We thus develop our method by summarizing the huge table into a small ruler with special scales. We also investigate the difference between Čobeljić et al. [4] and our methods. We hypothesize that if the difference is small, our method is comparable as that proposed by Čobeljić et al. [4] Thus our method can be more conveniently used for osteotomy in the clinical practice.

## Methods

The four-page table proposed by Čobeljić et al. indeed is a sine function table [4]. The table presents the correction chord distance as number, thus it takes much printed space. In order to simply the application of such a huge table to find out the proper chord distance in an osteotomy operation, we find it simpler to just show the correction chord distance as the proposed real distance for operation using a ruler instead of checking the attempted value from such a huge table. Thus we tried to summarize this huge numeric table into a small ruler.

First we define the row elements of the numeric table as the angles for correction osteotomy while the column elements as average diameters of the bone to be operated. According to Čobeljić et al. [4], the numeric table is made of the formula.

(1) *t *= 2r*sin(*α*/2)

where *t *means the chord distance for osteotomy; 2*r *means the average diameter of the bone; and *α *means the attempted osteotomy angle to be corrected during osteotomy.

**Figure 1:**
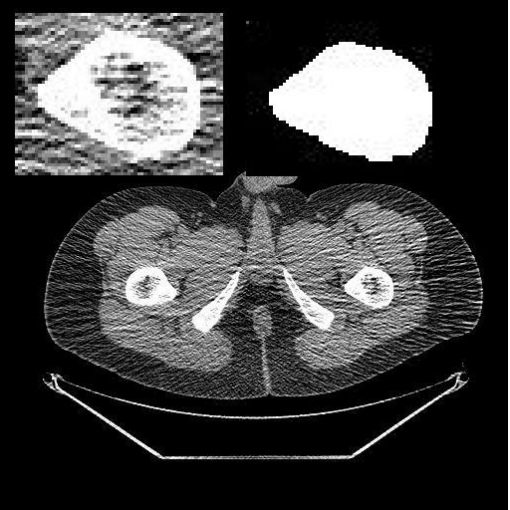
**The computer model of rotational osteotomy from CT**. The original CT slice is shown. (a) On the left top, we choose the interest region (around femur). (b) The right top shows the result of image processing. (c) The computer binarizes the picture, removes the isolated pixels, fills the medullary canal, and then calculate the gravity center and the errors.

The error of this method is due to mis-prediction of rotation radius, thus we build a computer model. We first get femur cross-section geometry data from CT (computer tomography) of pelvis below intertrochanter region. Then calculate the radius by method of Čobeljić et al., and then the actual distance from center of gravity to bondary. The differences are the cause of error. Then we calculate the error in percentage of these slices of CT (Fig. 1).

The average diameter is calculated by average of long and short axes of the bone (always ellipse in shape). In order to simply this calculation, we modify the formula.

(2) *t *= 0.5*(4*r*)*sin(*α*/2)

where 4*r *means the sum of long axis and short axis, that is twice of 2*r*.

We designed such a ruler and investigated the difference in various situations about the effects of geometrical morphology of the femur on the chord distance.

## Results

**Figure 2:**
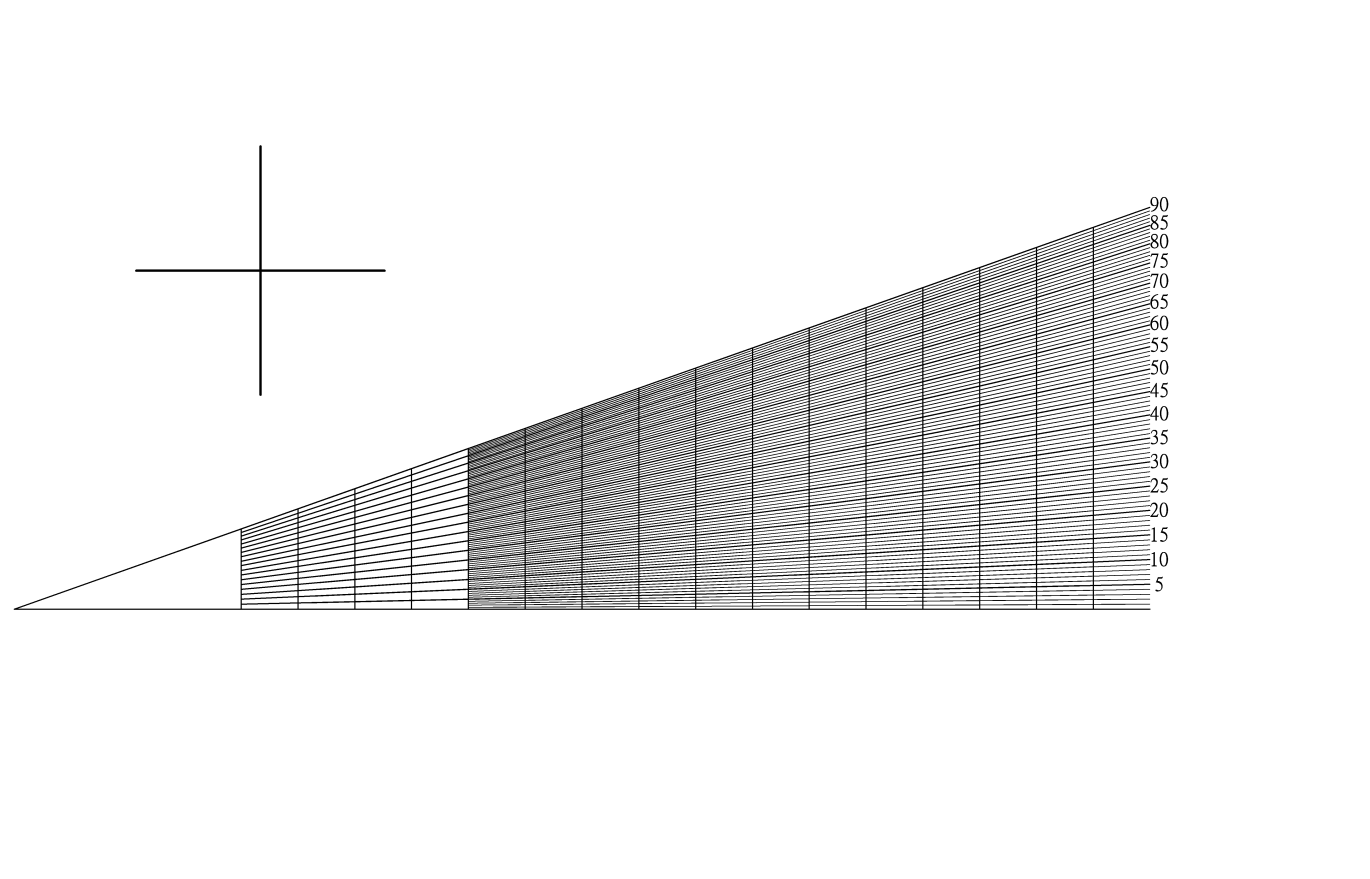
**The ruler**. We design our ruler for correction osteotomy by Formula (2). The cross on left upper site is for calibration. In any magnification, the two axes should be the same, otherwise the horizontal and vertical magnification powers are different and the ruler is imprecise.

**Figure 3:**
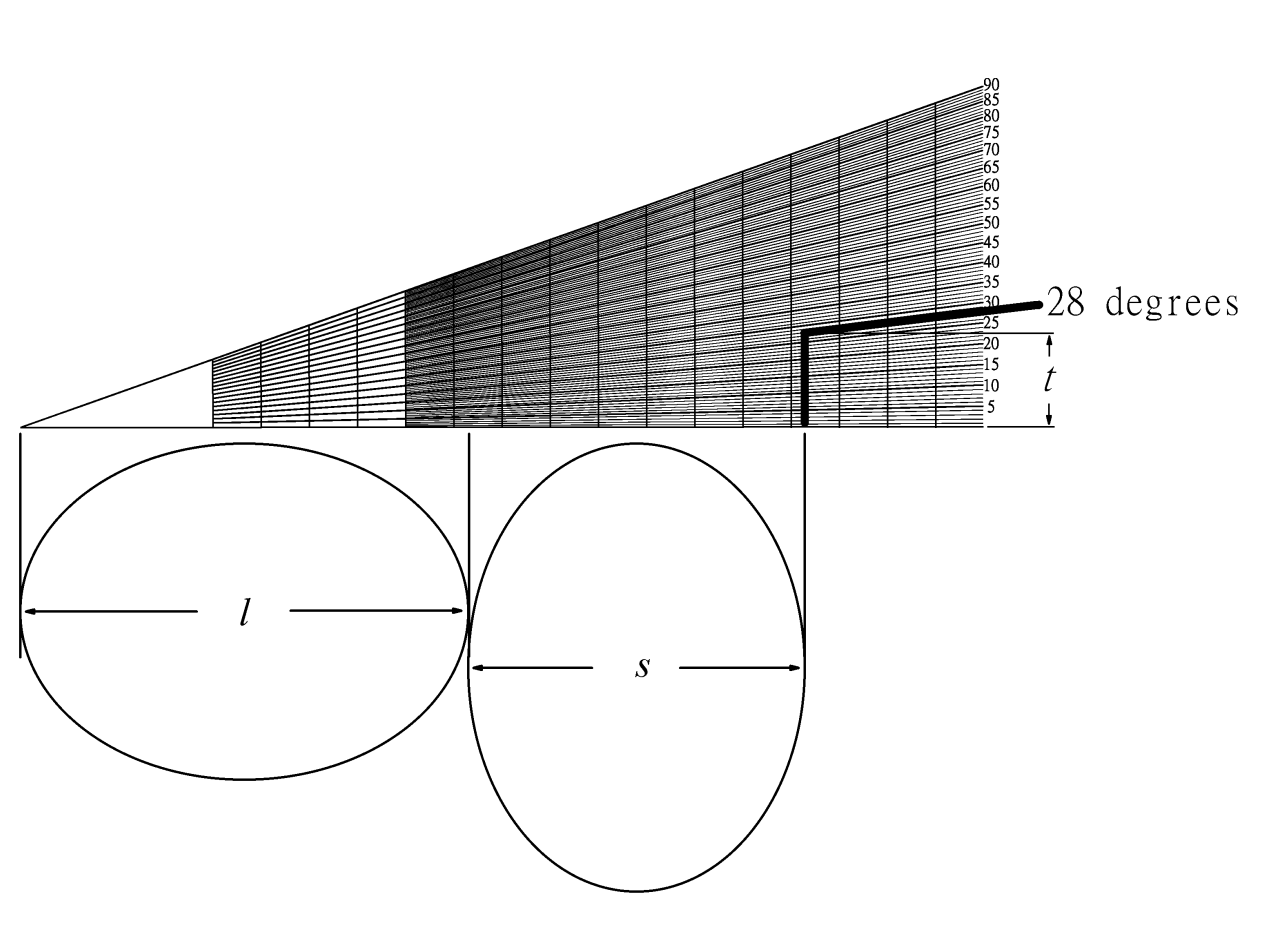
**Use of the ruler in ellipse**. Practically, we first make an osteotomy, then measure the long and short axes and mark on the ruler. The chord distance *t *can be read directly on the ruler at a proposed correction angle. In this example, we mark long and short axes, set the rotation angle = 28°. The chord distance can be read in the ruler directly.

**Figure 4:**
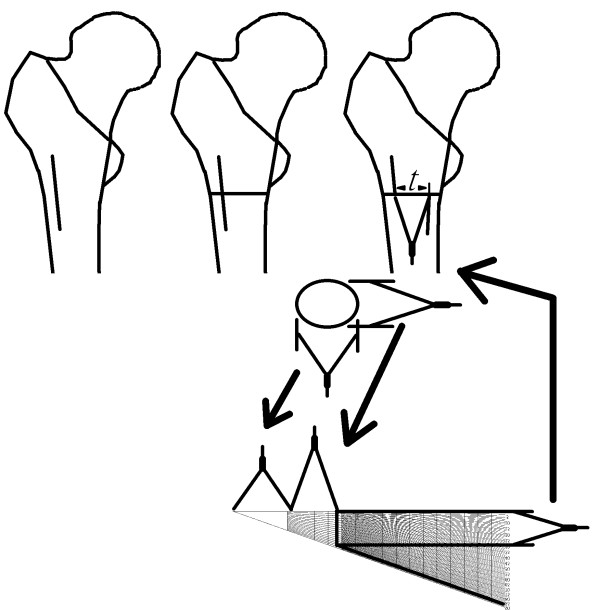
**Use of the ruler in proximal femoral rotational osteotomy**. This figure illustrates the chord distance *t *in a model of real osteotomy. In this situation, we use three pairs of compasses, one for the long axis, one for the short axis, and the last one for the chord distance *t*.

Our method basically is an improvement of the application for previously published four-table method. By the aforementioned formula (2) we developed our ruler for application in the correction osteotomy opertation. The horizontal axis is the distance of 4*r*, and the vertical axis is *t*. The ruler is shown in Fig. 2. For the practical use, the surgeon can first make the osteotomy, and then measure the long and short axes of the bone ends. Thereafter the long axis (*l*) and short axis (*s*) can be marked on the ruler sequentially, and the chord distance for osteotomy can be read by the proposed correction angle (Fig. 3, 4). Then the surgeons can precisely rotate the bone to meet the proposed chord distance followed by secure fixation (Fig. 4). Such a chord distance was determined by real measurement instead of the radiogram data.

To justify our protractor, we first verify Čobeljić et al. method by computer model (Fig. 1). Totally eight CT slices, 1558 bourdary points are collected. The error, which is estimated by calculating difference between real distance from center of gravity to boundary and the estimated radius, the error is between -37% to 16% (mean ± SD = -6% ± 9%). We think it is acceptable for these operations.

For further verification, we use a fixed the average diameter (2*r*) of 49 mm, and measure the chord distance for osteotomy for a range of osteotomy correction angles from 1° to 90°. Table 1 shows the summary of the difference between the correction chord distance proposed by the table from Čobeljić et al. [4] and our method from a range of correction from 1° to 90°. The chord distances using method proposed by Čobeljić et al. [4] and our ruler method for different chosen correction angles are listed in Table 1. The data for other degrees of osteotomy are not shown. The absolute difference between both methods ranges from 0 to 0.1 mm with a mean ± standard deviation (SD) of 0.03 ± 0.05 mm. Such a difference is acceptable during the real practice.

**Table 1:** Summary of the difference between the correction chord distance

**Correction angle**	**Chord diatance by Čobeljić et al.^3^**	**Chord distance by our ruler**	**Absolute Difference**
1°	0.4 mm	0.4 mm	0 mm
5°	2.1 mm	2.1 mm	0 mm
10°	4.3 mm	4.2 mm	0.1 mm
20°	8.5 mm	8.5 mm	0 mm
30°	12.7 mm	12.6 mm	0.1 mm
40°	16.8 mm	16.7 mm	0.1 mm
50°	20.7 mm	20.7 mm	0 mm
60°	24.9 mm	24.8 mm	0.1 mm
70°	28.1 mm	28.1 mm	0 mm
80°	31.5 mm	31.5 mm	0 mm
90°	34.6 mm	34.6 mm	0 mm
Mean			0.03 mm
Standard deviation			0.05 mm

Summary of the difference between the correction chord distance proposed by the table from Čobeljić et al. and our method for a range of correction from 1° to 90°. The data for other degrees of osteotomy are not shown.

Regarding the precision in the real practice, we afford an electrical version of our ruler in the real practice. The surgeon can magnify the ruler or on the computer screen to find out the final determined chord distance during an osteotomy procedure. Our invention is an image file, it can be printed in any size with propotional magnification. The magnification will not interfere its accuracy. In this study, we printed it as 5 cm-long size.

## Discussion

The rotational osteotomy is performed to correct the rotational deformity in the Orthopaedic practice, such as in the rotational deformity of the malunited fractures or to correct the excessive anteversion of proximal femur, etc. The clinical experience and comprehensive preoperative planning are important for the successful outcomes. Usually when we are doing rotational osteotomy, we must make the osteotomy perpendicular to the shaft, otherwise the rotational osteotomy may cause angulation. Furthermore, a convenient and precise apparatus may help much such a procedure and ensure a good expected result. Therefore we design such a ruler for the determination of the chord distance during a rotational osteotomy.

We verify our ruler at 49 mm diameter (2*r*), because this is the largest value mentioned in the table proposed by Čobeljić et al. [4] According to Formulas 1 and 2, the chord distance (*t*) is proportional to the diameter. If it works on the maximal diameter, it should also work on other diameter. We design this ruler and also measure the chord distance on the computer too. The original distance is in pixels and then is transformed to mm. Such a process can improve the precision.

**Figure 5:**
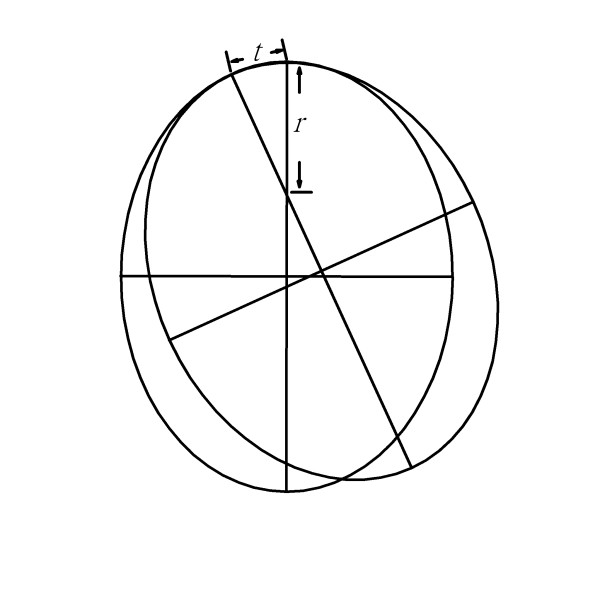
**Use of the ruler in least curve change strategy in small radius region**. Assuming that the cross-section of the bone is perfect ellipse, when we decide to fix the osteotomized bone with least curve change across bones, the chord distance (*t*) would be smaller that is accompanied by a larger regional curvature, i.e., a smaller regional radius (*r*).

**Figure 6:**
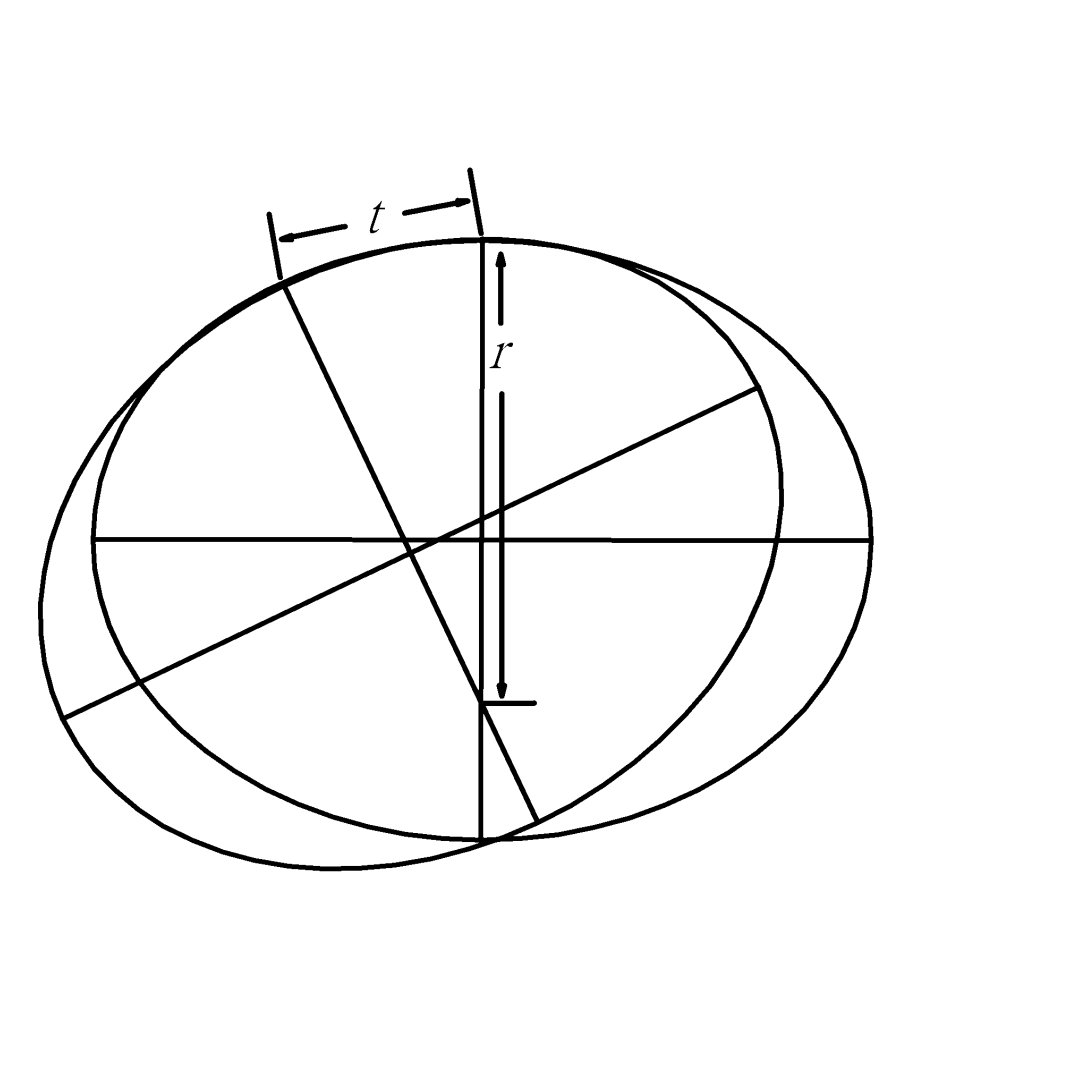
**Use of the ruler in least curve change strategy in large radius region**. The chord distance (*t*) would be larger that is accompanied by a smaller regional curvature, i.e., a larger regional radius (*r*).

**Figure 7:**
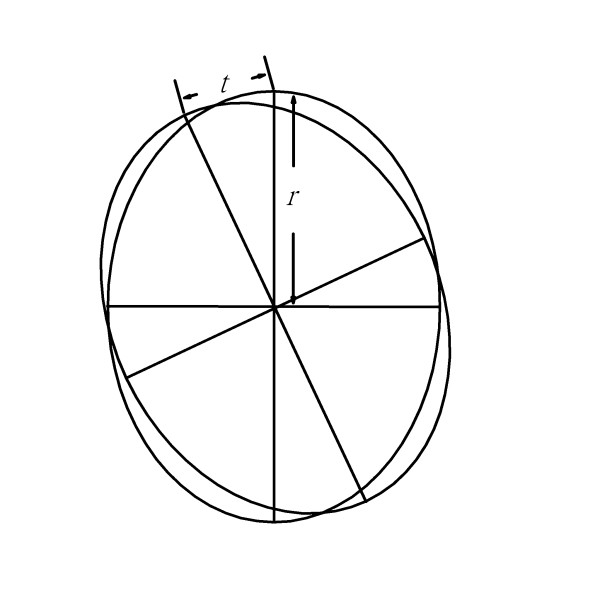
**Use of the ruler in rotation center strategy in large radius region**. Assuming that the cross-section of the bone is perfect ellipse, when we try to fix the osteotomized bone by using reference of the rotation center, the chord distance (*t*) is proportional to the distance to the center, the radius (*r*). When the radius is larger, the chord distance (*t*) is larger.

**Figure 8:**
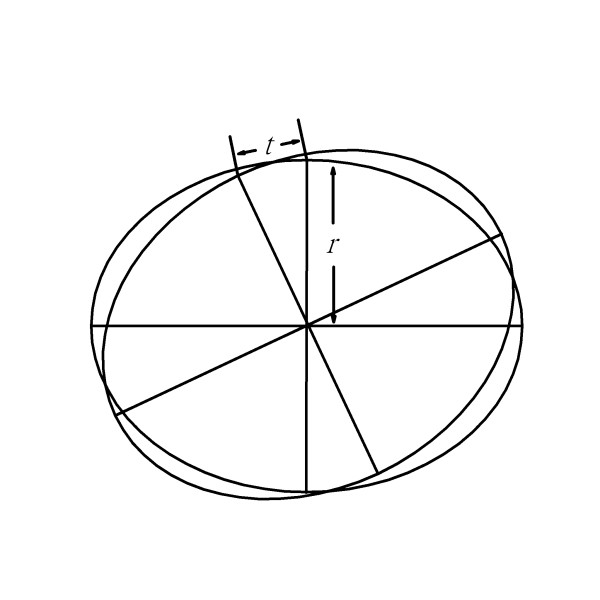
**Use of the ruler in rotation center strategy in small radius region**. When the radius is smaller, the chord distance (*t*) is smaller.

The ideal condition of the osteotomy procedure is the femur with a circular geometry. Equations 1 and 2 show good precision when the cross-section of the bone ends is circular. However, the long bone is usually not circular and somewhat elliptical. For such bones with non-circular ends, the precision for such a correction osteotomy might be influenced by the location of the fixation region. It depends on fixation strategy. If the surgeon tries to fix the osteotomized bone at site with least curve change across bones, the chord distance would be smaller that is accompanied by a larger regional curvature, i.e., small regional radius (Figs. 5, 6). In such strategy, the surgeon should choose plate to fix the osteotomized bone. Because the curvature is different around each segment of ellipses, the chord distance is also different. If the surgeon tries to fix the osteotomized bone by the reference using the rotation center, the chord distance (*t*) is proportional to the distance to the center (Figs. 7, 8). In such strategy, the alignment is better than the previous one, and the surgeon should choose intra-medullary nail or locking plate to fix the osteotomized bone. These are sources of errors. Therefore for the real practice, we recommend the use of a pair of compasses to measure the determined chord distance. In our computer model, with center rotation strategy, most of the errors are between 2% to -15%. The error is acceptable in such operation.

Despite these uncorrectable errors, our ruler precision is equal to the previous published table method by Čobeljić et al. [4] We propose such a ruler for more convenient use in the operation practice. Such a ruler (Fig. 2) can be made of a paper or stainless steel, and then we can use it on operation table after sterilization.

## Conclusion

We design a convenient ruler for application in the rotational osteotomy. Further clinical verification is warranted to justify it.

## Competing interests

This study was supported by the grant of NSC96-2320-B-087-001, Taiwan, ROC.

## Authors' contributions

The following authors have designed the study (CKL, RSY), gathered the data (TYW), analyzed the data (CKL, SMH, RSY), wrote the initial drafts (CKL), and ensure the accuracy of the data and analysis (CSF).

## Pre-publication history

The pre-publication history for this paper can be accessed here:



## References

[B1] Hoffer MM, Prietto C, Koffman M (1981). Supracondylar derotational osteotomy of the femur for internal rotation of the thigh in the cerebral palsied child. J Bone Joint Surg Am.

[B2] Lasserre J, Saint S, Girard (1966). [A simple geometric procedure applied to derotation osteotomy of the femur in children]. Ann Chir Infant.

[B3] Fait M, Janovec M (1976). [Subtrochanteric derotational femoral osteotomy (author's transl)]. Acta Chir Orthop Traumatol Cech.

[B4] Cobeljic G, Djoric I, Bajin Z, Despot B (2006). Femoral derotation osteotomy in cerebral palsy: precise determination by tables. Clin Orthop Relat Res.

